# Phage defence-system abundances vary across environments and increase with viral density

**DOI:** 10.1098/rstb.2024.0069

**Published:** 2025-09-04

**Authors:** Sean Meaden, Edze R. Westra, Peter C. Fineran

**Affiliations:** ^1^ Department of Biology, University of York, York YO10 5DD, UK; ^2^ York Biomedical Research Institute, University of York, York YO10 5DD, UK; ^3^ Environment and Sustainability Institute, Centre for Ecology and Conservation, University of Exeter, Penryn TR10 9FE, UK; ^4^ Department of Microbiology and Immunology, University of Otago, Dunedin 9016, New Zealand; ^5^ Genetics Otago, University of Otago, Dunedin 9016, New Zealand; ^6^ Bioprotection Aotearoa, University of Otago, Dunedin 9016, New Zealand; ^7^ Maurice Wilkins Centre for Molecular Biodiscovery, University of Otago, Dunedin 9016, New Zealand

**Keywords:** phage, phage defence, CRISPR

## Abstract

The defence systems bacteria use to protect themselves from their viruses are mechanistically and genetically diverse. Yet the ecological conditions that predict when defences are selected for remain unclear, as substantial variation in defence prevalence has been reported. Experimental work in simple communities suggests ecological factors can determine when specific defence systems are most beneficial, but applying these findings to complex communities has been challenging. Here, we use a comprehensive and environmentally balanced collection of metagenomes to survey the defence landscape across complex microbial communities. We also assess the association between the viral community and the prevalence of defence systems. We identify strong environmental effects in predicting overall defence abundance, with animal–host-associated environments and hot environments harbouring more defences overall. We also find a positive correlation between the density and diversity of viruses in the community and the abundance of defence systems. This study provides insights into the ecological factors that influence the composition and distribution of bacterial defence systems in complex microbial environments and outlines future directions for the study of defence-system ecology.

This article is part of the discussion meeting issue ‘The ecology and evolution of bacterial immune systems’.

## 1. Introduction

The immense diversity and complexity of the global virome have resulted in a multitude of ways that bacteria can defend themselves against viral infections. These defences span a range of mechanisms, including restriction modification (RM) and CRISPR systems, which recognize and cleave infecting viral genomes, while abortive infection (Abi) systems often trigger degradation of essential molecules of the host cell, preventing further spread of viral particles [1]. In recent years, many more types of defence systems have been identified, expanding the known ‘defensome’ [2], and this diversity of defences and virus-encoded counter-defences [3] suggests ongoing co-evolution and ecological significance. Many of these defence systems frequently co-occur within the same genome [4], with some combinations providing additional levels of defence through both additive and synergistic interactions [5–7]. In general, defence systems exhibit broad genetic and mechanistic diversity, encompassing those that degrade invading nucleic acids, such as RM and CRISPR-Cas, those that trigger cell death or dormancy, such as Abi systems like toxIN, or type-III CRISPR-Cas and CBASS, and those that exhibit numerous other mechanisms (reviewed in [8]). These diverse mechanisms may also be favoured under specific ecological scenarios (reviewed in [9]), for example systems that protect neighbouring cells may be more beneficial when environmental spatial structure is high [10]. However, linking these defence systems to environmental factors in complex environments is challenging, and the ecological drivers that shape their distributions in natural environments are less well understood. Finally, defence systems are frequently carried by mobile genetic elements (MGEs), allowing rapid mobilization into new hosts and facilitating competition between MGEs for shared hosts (reviewed in [11]).

Both theory and experiments have revealed potential drivers of the evolutionary ecology of defence systems (reviewed in [9]), but we lack a synthesis of this knowledge, and many open questions remain in complex microbial communities (reviewed in [12]). Abiotic factors generally determine microbial community composition (e.g. pH or salinity, [13]) and these vary substantially across environments, as shown by the Earth Microbiome Project [13]. In turn, biotic factors such as virus : microbe ratios, population sizes and interaction rates also vary across environments, and these biotic factors are likely to shape the composition of defence systems present [12]. For example, high relatedness between neighbouring bacteria has been shown to determine when Abi is a successful strategy [10], while the benefits of CRISPR-Cas systems outweigh receptor-based resistance when microbial biodiversity is high [14], whereas defence can be selected against in the presence of beneficial antibiotic-resistance-encoding plasmids [15]. As defence systems can be readily gained and lost, or inactivated, from bacterial genomes [15–22], their distributions may be optimized by ecological factors.

Genomic surveys of defence systems across bacterial and archaeal genomes have revealed some of these drivers of defence-system composition. Multiple studies have found strong effects of genome size on the abundance of defences [23,24]. Furthermore, by linking genomes to predicted traits, additional drivers can be inferred, such as aerobicity [25] and temperature [26] for CRISPR-Cas systems, temperature for RM systems and fast growth rates for overall defence abundance [27]. Expanding this approach to include genomes assembled from metagenomic data has shown that genomes from the gut environment carry substantially more defence systems than genomes from soils and the oceans, respectively [2], and that plant-associated bacteria have fewer defence systems than non-associated close relatives [28].

An additional factor predicted to shape the abundance and type of defence systems present in an environment is the density of viruses. Higher density would likely lead to more frequent infections and, in turn, stronger selection for defence systems. We previously identified that the abundance of CRISPR-Cas defence systems is both positively correlated with the abundance of viruses and varies widely across different microbial environments, with host-associated environments carrying more CRISPR than free-living environments [29]. Here, we collected a diverse range of metagenome assemblies from a public sequence data repository (MGnify, European Nucleotide Archive (ENA), [30]) and mined these assemblies for both defence systems and viral sequences. We then used coverage information as a proxy for the relative abundance of defence systems and viruses. We describe the distribution of defence systems across environments and variation in the total amount of defence systems, and link defence abundance to the abundance, or density, of viruses in each sample.

## 2. Methods

### (a) Data curation

We gathered a wide-ranging and standardized collection of metagenomes that had been processed using consistent methods. The MGnify database (ENA) was accessed via the API using the MGnifyR R package (https://github.com/EBI-Metagenomics/MGnifyR) on 14 June 2024. A search was conducted for all MGnify samples labelled as ‘assembly’ under the ‘experiment-type’ field and filtered to retain those processed using the MGnify pipeline v. 5.0. Metadata from the resulting 34 799 assemblies was collected with the getMetadata function from MGnifyR. Samples were then selected from categories in the ‘biome_string’ field that had >99 samples per group, with a requirement that only Illumina sequence data were included. The locations of the assemblies within the ENA database were located with the searchFile function of MGnifyR, and the resulting URLs were downloaded via a curl command on the University of York’s high-performance computing facility. The associated fastq data files for each assembly were downloaded using enaBrowserTools [31] and subsampled to 1 million reads per sample using seqtk [32]. Samples with fewer than 1 million reads were excluded from downstream analyses.

### (b) Metagenome community composition and diversity

From the subsampled 1 million reads from each sample, 500 000 were taxonomically profiled using Kraken 2 with the Kraken 2 standard database [33]. Taxonomic groups that made up less than 0.01% of the relative abundance were removed. Diversity at the genus level was calculated, and principal coordinate analysis (PCoA) clustering was performed using the R package vegan [34].

### (c) Defence-system search

Contigs were first annotated for coding sequences using Prodigal (default settings, translation table 11) [35]. Comparison on a subset of samples found that when the ‘meta’ mode was used, the results were almost identical. Assemblies shorter than 100 kbp were excluded. Contigs were searched for defence systems using PADLOC ([36], v. 2.0) with the PADLOC database (v. 2.0). Defences labelled as ‘DMS_other’, ‘dXTPase’, ‘PDC’, ‘HEC’ and ‘VSPR’ were discarded as these are either non-defence, not experimentally verified or unpublished predicted defence systems at the time of analysis, although some HEC systems have been subsequently verified [37]. We also used DefenseFinder to identify defence systems and compared these results with our PADLOC results. We found that the number of defences identified by each tool correlated very strongly and that these correlations were consistent across environments (electronic supplementary material, figure S1), although PADLOC recovered slightly more systems. Benchmarking between tools and for specific systems has been previously described for complete genomes [38], and we solely used the PADLOC outputs for downstream analysis. Assemblies were searched for CRISPR arrays with metaCRT using default parameters [39].

### (d) Viral sequence search

Assemblies were searched for viral sequences using the geNomad pipeline [40]. The tool uses a dual strategy approach of both marker-based and alignment-free classification. Sequences (contigs in this study) are either used for gene prediction and annotation against a reference catalogue of markers or grouped based on sequence similarity using a neural network model. These results are then aggregated for each sequence to provide a classification as either plasmid, viral or chromosomal. Full details of the methods and construction of the marker protein profiles are available in [40]. During post-processing, sequences shorter than 1 kb and those lacking any ‘hallmark’ (as classified by geNomad) viral genes were discarded. Sequences were classified as chromosomal, plasmid or phage based on the maximum score ascribed by geNomad. The genomic locations of defences were identified using the geNomad predictions for chromosome, phage, plasmid or prophage, again using the maximum score.

### (e) Abundance estimation

The associated sequencing reads were mapped to the assemblies with bwa ([41], v. 0.7.17) and processed with SAMtools ([42], v. 1.9). The resulting alignment files (BAM format) were used to calculate coverage and read recruitment values for all contigs in each assembly using coverM with the ‘contig’ command (v. 0.7.0) and the ‘metabat’ and ‘count’ methods, respectively. The contigs identified from the defence system and viral sequence searches were then extracted from the coverM results tables and used for downstream analysis. These per-contig abundance files were then merged with the PADLOC results, with one count value recorded per defence system-type per contig. In cases where one contig carried multiple defence systems, the count value was recorded for each defence type. Notably, of all the contigs identified as carrying a defence system, >95% carried a single defence system type, likely owing to the highly fragmented nature of the assemblies (electronic supplementary material, figure S2). The total defence abundance per sample was calculated by the sum of reads mapping to contigs carrying each defence system. We opted here to focus on measuring the abundance of defence systems and therefore count multiple unique defence systems on a single contig independently, effectively ‘double counting’ contig read recruitment if it carried multiple unique defences. In practice, this represented <5% of all contigs owing to the fragmented nature of the assemblies (4% carrying two systems, 0.4% carrying three systems and 0.5% carrying four or more systems). Count data were also obtained using the same mapping-based approach for those contigs predicted to be of viral origin: abundance tables were extracted from the coverM results tables based on the geNomad predictions and collated into a master file containing the results from all samples.

### (f) Sequencing effort and eukaryotic contamination controls

We assessed the effect of sequencing depth by first collecting the associated fastq data and counting the number of reads. We also assessed eukaryotic (human) DNA contamination, predicted from the Kraken 2 analysis, and found higher levels of eukaryotic DNA associated with human-associated samples (electronic supplementary material, figure S3). When analysis was restricted to samples with <50% of classified reads being of eukaryotic origin and with the inclusion of assembly N50 and original sequencing depth, the results were qualitatively the same. There remained a strong environmental effect on defence abundance and a consistent correlation between viral abundance and defence abundance.

### (g) Taxonomic identification of plant-derived defence systems

We observed a high number of argonaute and Tiamat systems in the plant-associated samples. To assess the origin of these defence systems, we extracted the corresponding contigs, on which these defences were located, and assigned a taxonomic classification using the MMSeqs2 taxonomy pipeline against the NCBI non-redundant (nr) nucleotide database (downloaded 25 August 2024). Contig classifications based on the last common ancestor were visualized in R.

### (h) Statistical analysis

The effect of environment on total defence abundance was assessed using a generalised linear model (GLM) with a ‘quasi-Poisson’ error structure. A measure of assembly fragmentation (N50 value) was included in the model, as preliminary analysis found a significant relationship between N50 value and defence abundance. Significance was assessed with ANOVA by comparison against a null model with the environment term removed.

The effect of the environment on defence-system composition was assessed by first converting the results to a sample by defence-system abundance matrix. Permutational ANOVA was then applied using the ‘adonis2’ function from the R package vegan. This function converts the abundance matrix to Bray–Curtis distances of dissimilarity between samples and applies a permutational ANOVA. In total, 999 permutations were used. Ordinations were conducted using the ‘NMDS’ and ‘pco’ functions in vegan based on Bray–Curtis dissimilarity values. Taxonomic dissimilarity scores were extracted from the Kraken 2 analysis and compared with the defence composition dissimilarity scores using Spearman’s correlation.

Correlations between defence abundance and viral abundance were assessed using a linear model including N50 as a covariate and a Gaussian error structure. Defence abundance and viral abundance counts were log_10_-transformed to improve model fit, and model residuals were assessed visually. Significance was assessed by ANOVA against a null model with viral abundance removed. We repeated the above-mentioned statistical tests on a restricted dataset that had the following criteria: all samples had <50% of reads classified as eukaryotic, and both the assembly N50 value and the read count of the original samples were included in the model. This aimed to account for assembly fragmentation and the sequencing depth, based on the assumption that all the data were used to generate each of the assemblies.

## 3. Results

### (a) Defence abundance, diversity and composition vary across environmental categories

**Figure 1.**
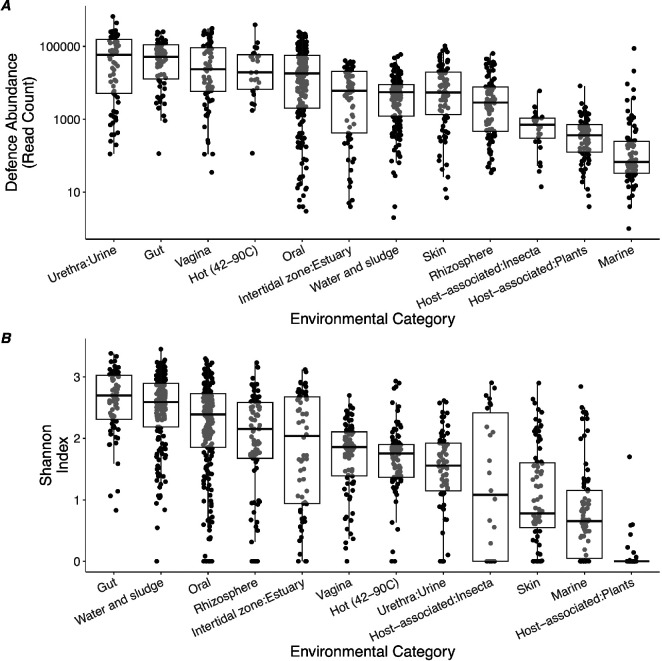
Defence abundance and diversity vary across microbial environments. (*A*) Points represent individual metagenomes grouped by biome metadata categories provided from the European Nucleotide Archive (ENA). Defence systems were identified from metagenomic contigs using PADLOC [36], a standardized subsample of 1 million reads was mapped to each assembly, and the counts of contigs carrying defence systems were summed to count total defence per sample (>95% of contigs had a single defence system). Boxplots show median values, first and third quartiles and whiskers extend to smallest and largest values, excluding outliers. (*B*) Boxplots showing defence diversity (Shannon index) for each environment. Points represent individual metagenomic assemblies. Shannon’s diversity index was calculated from a subsample of 1 million reads mapped to contigs carrying defence systems.

The discovery of novel defence systems continues at pace, but our knowledge of the ultimate drivers of their evolution and ecology is lacking. Here, we collated an ecologically diverse collection of 1075 metagenomes from 12 environments. We then surveyed the defence repertoire and abundances using a homologue-search-based tool [36] to assess which environments carry the most defences and the composition of defence systems in those environments. We found that the total abundance of defence-carrying contigs significantly varied among environments (*F*_11,1037_ = 46.0, *p* < 0.0001), suggesting that environmental conditions strongly affect when defence systems are optimal. Notably, animal-host-associated environments (urethra, gut, vagina, oral and skin) were highest in overall defence abundance, with hot environments similarly high in defence abundance (figure 1).

**Figure 2.**
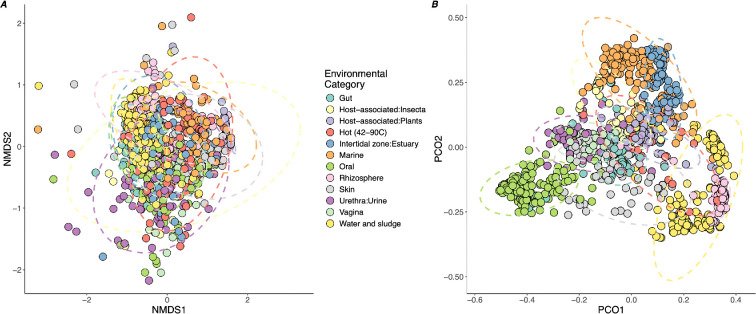
Defence and taxonomic composition variation between environments. Ordinations of metagenomic samples based on similarity in defence composition (*A*) or taxonomic similarity (*B*). Groups that cluster together share more defence systems or prokaryotic taxa, respectively. Points represent individual metagenomic assemblies, ellipses represent 95% confidence intervals for a multivariate *t*-distribution, and colours show the environment sampled. (*A*) NMDS analysis was performed on a defence-system abundance table constructed from a subsample of 1 million reads mapped to contigs carrying defence systems. (*B*) A subsample of 500 000 reads was classified with Kraken 2, and the frequency of each genus was obtained. Both ordinations use Bray–Curtis dissimilarity scores calculated from their respective abundance tables.

We then calculated the diversity of defences (Shannon’s index) for each sample, which also varied significantly with environment (*F*_11,1064_ = 75.2, *p* < 0.0001; figure 1). Overall, defence diversity was correlated with defence abundance (*F*_1,987_ = 719.9, *p* < 0.0001); however, there were minor differences in the ranking of the environmental categories. Water and sludge, estuary and soil (rhizosphere) environments had similarly high defence diversity to the host-associated samples, despite lower defence abundances. We also observed substantial variation in the abundance and distribution of specific defences, with a notably sparse distribution, meaning that most defences were rare or absent in the majority of samples (electronic supplementary material, figure S4). To assess the role of the environment in shaping the defence composition, we applied a permutational ANOVA to a Bray–Curtis matrix of dissimilarity between samples using environment as a fixed effect. We found a significant effect of environment (*F*_11,1133_ = 23.0, *p* < 0.001) with an *R*^2^ value of 0.18. Despite this significant effect, inspection of NMDS ordinations showed substantial overlap between groups (figure 2*A*), preventing accurate prediction of defence composition from environmental information alone. Importantly, taxonomic profiling of the same samples did show strong clustering by environment (figure 2*B*), and we observed a substantial mismatch between overall community diversity and defence diversity (electronic supplementary material, figure S5; figure 1*B*). To assess the contribution of taxonomic composition to defence composition, we assessed the correlation between these pairwise distance scores for all samples. We found a significant but weak association between defence composition and taxonomic composition (Spearman’s *ρ* (*n* = 427 286): 0.223, *p* < 0.0001). The pan-immune hypothesis predicts that insufficient defences can be carried on a single genome for lasting resistance, but novel defences can be acquired or lost to a wider pool [43]. Our results are broadly consistent with this view in that differences in defence composition are unlikely to be driven solely by bacterial taxonomic effects, presumably owing to frequent gain and loss via horizontal gene transfer.

### (b) Defence abundance correlates with viral abundance across environments

**Figure 3.**
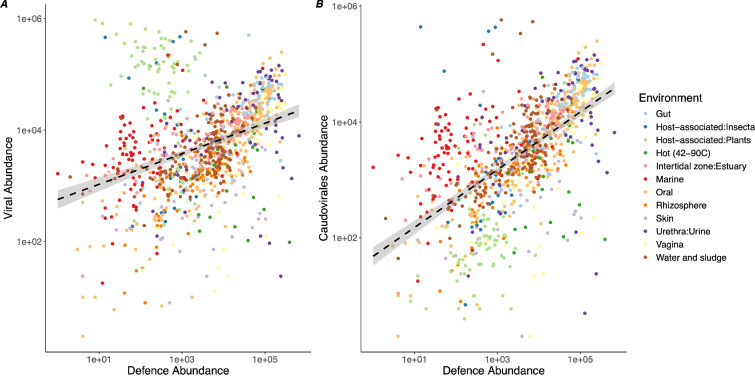
Defence abundance correlates with viral abundance. Metagenomic assemblies were mined for phage defence systems and viral sequences. Coverage values were collected from a subsample of 1 million reads per sample. Viral abundance represents the sum of all read counts for virally classified contigs; defence-system abundance represents the sum of reads mapping to contigs carrying defence systems. The dashed lines represent linear models, and the shaded area represents 95% confidence intervals. (*A*) shows the sum of all viral contig read counts, while (*B*) shows a subset of counts restricted to contigs annotated as the dsDNA tailed phage group Caudoviricetes.

In addition to abiotic factors, biotic factors (and in particular the viral community composition) are likely to influence the abundance of phage defence systems. To assess this, we first estimated the abundance of viruses in the sample based on coverage of viral contigs from a standardized subset of reads for each sample (1 million). These estimates, therefore, do not represent absolute viral abundances, which likely vary substantially between environments, but measure viral abundance relative to microbial DNA sequences as the majority of reads are of bacterial origin. We found a significant correlation between overall defence abundance and viral abundance (*F*_1,997_ = 115, *p* < 0.0001; figure 3), suggesting that the density of viruses is a strong selective force for phage defence systems. As an additional test, we restricted the analysis to just those viruses annotated as Caudoviricetes as a way of excluding effects caused by non-phage environmental viruses. In this case, we also observed a significant positive correlation with overall defence abundance and Caudoviricetes abundance (*F*_1,978_ = 135.0, *p* < 0.0001; figure 3). Unsurprisingly, viral diversity and viral abundances were strongly correlated (Pearson correlation coefficient: 0.39, *p* < 0.0001). We refer herein to viral abundance, but note that the accompanying viral diversity may also be contributing to the observed effects. We also identified the predicted genomic context of each defence system to assess the proportions of defences carried on MGEs. We found that 54% of defences were located on chromosomal contigs, 32% on plasmid contigs, 13% on viral contigs and 0.3% on integrated prophages (electronic supplementary material, figure S6). We note that many of the viral contigs likely represent fragments of prophages, and the low number of integrated prophages results from the scarcity of fully intact prophage genomes complete with chromosomal flanks owing to fragmented metagenomic assemblies.

### (c) CRISPR array abundance correlates with viral abundance across environments

Our previous work assessed the abundance of CRISPR defence systems across environments. We aimed to use a similar approach across the wider pool of samples here, again focusing on CRISPR arrays rather than effector proteins. By focusing on CRISPR arrays, we aimed to mitigate the difficulties of detecting multi-gene systems in fragmented, short contig, metagenomic data. As before, we found a positive correlation between CRISPR array abundance and viral abundance (*F*_1,911_ = 4.33, *p* = 0.04) and a similar relationship when we restricted the analysis to Caudoviricetes abundances (*F*_1,896_ = 9.56, *p* < 0.01). We also assessed the origins of the arrays and found that similar numbers of arrays were predicted to be located on chromosomes (40.2%) and viral contigs (44.7%), which include prophages, with fewer predicted on plasmids (15.1%). When we repeated the correlations between CRISPR abundance and viral abundance for these subsets of the data, we found significant positive correlations between CRISPR abundance and viral abundance for the chromosomal- (*F*_1,838_ = 208.0, *p* < 0.0001) and plasmid- (*F*_1,697_ = 93.7, *p* < 0.0001) derived array abundances, but not for the virally derived arrays (*F*_1,814_ = 0.94, *p* = 0.99). Taken together, these results suggest that the selective forces that determine when CRISPR is beneficial may differ between bacteria and plasmids versus phages, potentially owing to stronger selection for streamlined genomes in viruses.

### (d) Reduced defence-system prevalence in plant-associated environments

While the abundance of defences generally correlated with viral abundance across environments, the plant metagenomes used in this study were obvious outliers, harbouring a highly reduced number of defence systems and elevated viral abundance (figure 3*A*). Plant microbiome samples typically suffer from high levels of plant DNA contamination owing to sampling techniques [44]; however, recent work has also found phage defence systems to be underrepresented in plant environments [28]. The defence systems most abundant in the plant samples in our dataset were PD-T4, argonautes and Tiamat. Argonautes are well characterized plant immune effectors in RNA silencing immunity [45] and it is therefore unsurprising that they are prevalent, if the metagenome is largely plant–host derived. To assess the taxonomic origins of argonaute and Tiamat systems, we extracted the contigs containing these systems and classified them using the NCBI non-redundant nucleotide database. For the plant-associated metagenomes, 86% of argonaute and 100% of Tiamat systems were located on plant genome sequences (electronic supplementary material, figure S7). These observations of plant-derived sequences are supported by the presence of many viral sequences annotated as RNA viruses, despite the data being of metagenomic origin, which can occur as a result of endogenous retroviruses integrated into the plant genome. Despite these technical aspects of plant microbiome sampling, further work must assess the biological reasons for an underrepresentation of phage defence systems in plant environments [28].

## 4. Discussion

Previous work has identified variation in both the frequency and types of phage defence systems found across different natural environments using metagenome assembled genomes [2]. Here, we search the full metagenome, at contig level, for both defence systems and viral sequences to conduct a survey of the defence systems present in a broader range of environments using publicly available metagenomic data. We have previously shown that the abundance of a specific group of defence systems, CRISPR-Cas, is strongly correlated with the relative abundance of viruses present in the environment [29]. We found a strong correlation between the total abundance of defence systems and viral abundance, consistent with the notion that the viral community is a strong selective force for the acquisition and retention of phage defence systems.

In agreement with other work, gut samples harboured a greater abundance of defence systems than soil or marine environments, respectively [2]. Notably, five out of the top six environments for viral abundance are human-host associated, with the exception being samples derived from environments of 42°C or higher (such as hot-spring thermal environments). Hot-spring environments are suggested to be a hotspot for virus-defence systems owing to higher costs associated with mutations, via reduced protein stability, in turn reducing viral diversity and the potential for defence evasion [46]. By contrast, bacteria and archaea from mesophilic environments may be more able to tolerate a wider range of mutations, requiring more robust resistance mechanisms, such as mutation of surface receptors and subsequent phage resistance. Indeed, recent work suggests surface receptor variation can be a stronger predictor of successful phage infection than intracellular defence systems [47]. Our results are consistent with the notion of human-host environments providing a resource-rich environment for microbes, in turn hosting a greater density of viruses and selection for defence systems.

Interestingly, marine environments appear to be particularly depleted in defence systems. Although the marine environment is predicted to have a high daily viral lysis, which is consistent with strong selection for defence, the virus-to-microbe ratio is low [12]. Furthermore, infection assays of culturable marine microbes found low predation pressures likely owing to low encounter rates [48]. In addition, both the dominant marine clade, SAR11, and the marine cyanobacterium *Prochlorococcus* have undergone genome reduction [49], presumably reducing the capacity for carrying diverse intracellular defence systems. The low overall density of defences we observed is consistent with relatively weak selection from phages, and the typically low nutrient conditions may make the carriage of defence systems costly compared with selection for reduced genome sizes. In support of this conclusion, analysis of cyanobacteria and their phages found far more defence systems in freshwater genomes, which typically have higher nutrient availability, than in those from marine environments [50]. In contrast to human-host-associated samples, insect-associated samples also carried far fewer defences. It has been observed previously that insect-associated bacterial genomes have few or no defences [23,51], likely due to either the general genome reduction processes that occur in intracellular insect symbionts [52] or reduced phage predation in the endosymbiotic environment (discussed in [53]).

Plant-associated samples also carried far fewer phage defence systems than human-host-associated samples. The phyllosphere is typically low in carbon and nitrogen and relatively oligotrophic [54], again potentially increasing the costs of phage defence-system carriage. Recent work has found that plant-associated bacteria are depleted in defence systems relative to non-plant-associated relatives [28]. However, our results may also be partially due to technical artefacts of sampling plant tissue and the typically high levels of host contamination. Specifically, in our dataset, we found argonautes to be the most abundant defence, which are common in plant genomes, functioning as RNA interference (RNAi) effectors [45]. The viral community was also consistent with this conclusion, as although Caudoviricetes was the most frequently identified viral group, we found many groups of Riboviria. These are RNA viruses capable of integrating into plant host genomes as endogenous retroviruses. Surprisingly, along with argonautes, we also identified a high frequency of the Tiamat and PD-T4-6 defence systems. When we identified the origins of the Tiamat and argonaute systems, these were almost exclusively from plant sequences in the plant samples, versus a wide range of bacteria in the other environmental samples (electronic supplementary material, figure S7). Further work is needed to assess the reasons why the plant-associated metagenomes were so depleted in defence systems [28] and the extent of defence conservation across domains of life [55].

By mining existing metagenomic assemblies, our results may be skewed towards viruses that are enclosed within a cell, either as prophages or undergoing active replication [56], although some viral particles will be present, as bulk metagenomes typically contain the most abundant viral genomes [57]. Yet experimental work has shown that the extracellular viromic fraction in an environment can change quickly, both temporally and spatially [57]. It is also challenging with metagenomic data to disentangle viral diversity and viral abundance, as these factors strongly covary. We suggest experimental work in this area will yield valuable insights into the ultimate driver of defence-system composition. Theory suggests that increased viral mutation rates limit the effectiveness of adaptive immunity [46], but further studies are needed. In addition, we focus entirely on DNA viruses, but RNA viruses are common [58,59], albeit less well studied. Assessing patterns of defence prevalence in light of RNA virus abundances and integrating spatial and temporal information will be important future work. We also cannot rule out some biases in our search strategy and currently available methods, as most defence systems, including those derived from a wide range of non-model bacteria and archaea, have been functionally validated in a limited number of model organisms [60,61]. It is possible that some incompatibility between the defence-system origin host and the taxa chosen as model organisms creates biases in defence-system discovery. We suggest that future efforts to identify viruses and defence systems from non-model organisms and environments will greatly expand our understanding of the role of the environment in shaping these interactions. We also note that our results are skewed towards smaller defence systems, with fewer core genes, owing to the fragmented nature of typical metagenomic assemblies (electronic supplementary material, figure S8). Longer, multi-gene defence systems are more likely to span multiple contigs in the assembly and will therefore not be detected by defence identification tools, which rely on finding core genes and/or a minimum number of genes depending on the system. Finally, metagenomic assemblies are rarely exhaustive and likely represent the most abundant organisms in an environment; as such, our defence-system survey is representative of those that exist in the most abundant species, and many others almost certainly exist in those environments at lower frequencies. Future efforts must focus on more contiguous assemblies derived from long-read sequencing, which will be vital for more fine-scale metagenomic analysis.

We found a consistent positive correlation between defence abundance and viral abundance; however, viruses and other MGEs are well known to carry defence systems. Our analysis found that up to 46% of defences were predicted to be located on MGEs. Therefore, this high percentage of MGE-associated defence systems may be driving the observed correlations. This leads to two possible interpretations: firstly, that defence accumulation on MGEs is a neutral process or ‘lottery’ effect and will occur more frequently when MGE abundances are high, or secondly, that when MGE abundances are high, there is greater competition between MGEs for susceptible hosts, requiring more defence systems to target competitors [11,62]. We suggest that experimental work in this area will yield valuable insights into natural microbial community dynamics of MGE competition and the interplay with defence systems.

Overall, we have identified wide variation in defence abundances across microbial environments and the density of viruses as a likely driver of selection for defence systems. Despite differences in defence abundance, there were minor differences in defence-system composition across environments. This was surprising given the strong clustering of samples at the taxonomic level (figure 2) and suggests that while the environment predicts the overall abundance of defence, it does not strongly shape the defence composition. Clearly, further work that integrates community ecology and metagenomic analysis is needed to assess whether the accumulation of specific defences is a stochastic process or determined by other, unmeasured parameters. We also predict that further work may identify the ultimate selective forces acting on individual defence systems. As ever more defence systems are discovered, we anticipate future studies will focus on the individual ecology of system types and classes of defence [8] and anti-defence [3], potentially identifying environmental hotspots that would allow targeted search strategies for defence discovery.

## Data Availability

All data used are publicly available from the MGnify database. A list of accession numbers can be found in the electronic supplementary material, file S1. Code used for the analysis is available at [63]. Supplementary material is available online [64].
